# Diffuse large B-cell lymphoma involving multiple immunoprivileged sites presenting as painful ophthalmoplegia: A case report

**DOI:** 10.1097/MD.0000000000032695

**Published:** 2023-01-20

**Authors:** Yimei Zhang, Zhang Jianfeng

**Affiliations:** a Department of Neurology, Jinshan Hospital Afliated to Fudan University, Jinshan District, Shanghai, China.

**Keywords:** case report, diffuse large B-cell lymphoma, immune privileged organ, primary central nervous system lymphoma, testicular lymphoma

## Abstract

Primary diffuse large B-cell lymphoma (DLBCL) is the most common type of non-Hodgkin’s lymphoma. It is very rare to find lymphoma in immune -privileged sitesat the same time. We report a case of rapidly progressing DLBCL involving multiple sites in the central nervous system, testis, and orbit.

**Patient concerns:**

A 57-years-old man developed diplopia 1 month ago with severe right periorbital pain and right eyelid ptosis. He developed dysphagia 1 week ago and hoarseness 5 days ago.

**Diagnoses:**

The pathology of the left testicle confirmed DLBCL. Immunohistochemical analysis showed that CD20, CD79α, multiple myeloma oncogene-1, BCL2, BCL6 atypical lymphocyte aggregation was positive. Positron emission tomography reveals DLBCL involving the central nervous system, testes, eyes, and other parts of the body.

**Interventions:**

We administered glucocorticoids for pre-chemotherapy treatment, but the patient’s condition progressed quickly and was generally poor. The patient’s family decided to discharge automatically.

**Outcomes:**

Two weeks after he was discharged, we called for a follow-up visit and were told the patient had died.

**Lessons:**

Atypical clinical symptoms of the disease often confuse the doctor’s diagnosis. Adequate examination should be performed before glucocorticoid treatment in order to avoid obscuring the true condition. In some rare diseases, early use of PET-CT may lead to early detection.

## 1. Introduction

Painful ophthalmoplegia syndrome, also known as Tolosa Hunt syndrome (THS), was first reported by Tolosa and Hunt in 1954 to 1961, and the earliest diagnostic criteria were proposed.^[1,2]^ THS is a disorder caused by nonspecific granulomatous inflammation of the cavernous sinuses, supraorbital fissure, or orbital apex, the cause of which is unclear. Typical patients with THS present with severe pain in the retro-orbital and head on 1 side, with often ipsilateral III, IV, VI cranial nerves causing ophthalmoplegia, some of which can involve the ocular and maxillary branches of the fifth nerve. This disease is effective for glucocorticoid therapy.^[3]^

Diffuse large B-cell lymphoma accounts for about 30% to 35% of non-Hodgkin’s lymphoma. Diffuse large B-cell lymphoma (DLBCL) is prone to recurrence after chemotherapy and it is one of the refractory tumors.^[4]^ About 30% of patients present with extranodal onset, common sites of origin are the digestive system, skin and soft tissue, skeletal system, or urogenital system. But in advanced stages, as the disease spreads to the bone marrow, pleura, peritoneum, and central nervous system, the site of the primary disease sometimes becomes unclear.^[4,5]^ DLBCL is a heterogeneous disease that can be identified by 2 molecular subtypes, namely germinal center B cell-like DLBCL (GCB) and activated B cell-like DLBCL (ABC), and patients with GCB subtype usually have a better prognosis than those with ABC subtype.^[6]^

The central nervous system (CNS), eyes, and testis are considered immune-privileged sites where lymphocytic cells may escape immune surveillance and thus be protected from damage by the immune system and chemotherapy. This also indicates the complexity and difficulty of lymphoma in these areas.^[7,8]^ Primary diffuse large B cell lymphoma of the CNS (PCNSL) is a rare and invasive type of non-Hodgkin lymphoma, mostly DLBCL. This disease has a high degree of malignancy and a low clinical cure rate, accounting for about 3% to 5% of primary brain tumors. 95% of the disease mainly involves the brain, eyes, meninges and spinal cord, rarely affecting the whole body.^[9]^ Primary testicular lymphoma (PTL) is the most common testicular malignancy in men over 60 years of age, with a high rate of late recurrence in other parts of the body, especially in immune-privileged areas. Primary vitreoretinal lymphoma is classified as a subtype of PCNSL, and about 1-third of primary vitreoretinal lymphoma patients present with PCNSL, most of whom will develop PCNSL.^[10]^

We report a case of rapidly progressing DLBCL involving multiple sites in the CNS, testis, and orbit. The disease begins as a clinical symptom of unilateral painful ophthalmoplegia.

## 2. Case report

A 57-years-old man presented to the Department of Neurology, Jinshan Hospital affiliated to Fudan University, with diplopia and right eye pain for 1 month, dysphagia for 1 week, and hoarseness for 5 days. The patient presented with diplopia about a month ago, manifested as left and right double vision, without blurred vision. The patient presented with severe periorbital distention and ptosis of the right eyelid. There was no significant difference between morning and evening symptoms. At the local hospital, doctors conducted a series of tests. At the local hospital, doctors conducted a series of tests. Head MRI suggested a new cerebral infarction near the anterior corner of bilateral ventricles. There are multiple ischemic foci in bilateral frontal parietal lobe and right basal ganglia. The patient’s symptoms did not improve significantly after oral administration of pyridinstigmine bromide. The myasthenia gravis antibody test was negative. The patient was transferred to our hospital for DSA examination, and no aneurysm or other vascular malformation that might cause oculomotor nerve palsy was found. Considering the patient’s painful ophthalmoplegia, dexamethasone was given as symptomatic treatment, and Omeprazole was given as a stomach protection treatment at the same time. After a series of treatments, the patient’s diplopia, impaired eye movement, and right orbital pain were better than before and he was discharged from the hospital. The patient developed dysphagia and hoarseness 1 week ago, and reappeared 2 days ago with severe right periorbital pain, accompanied by nausea and vomiting. The patient had a history of hypertension for 5 years, and the highest systolic blood pressure was 170 mm Hg. The blood pressure was controlled by oral administration of irbesartan and hydrochlorothiazide. The patient was a former carpenter who lived with his wife in a small city and had a healthy daughter. He has a smoking history of more than 30 years, about 10 cigarettes per day, and has not smoked for nearly 2 months. He denies the history of alcoholism.

**Figure 1:**
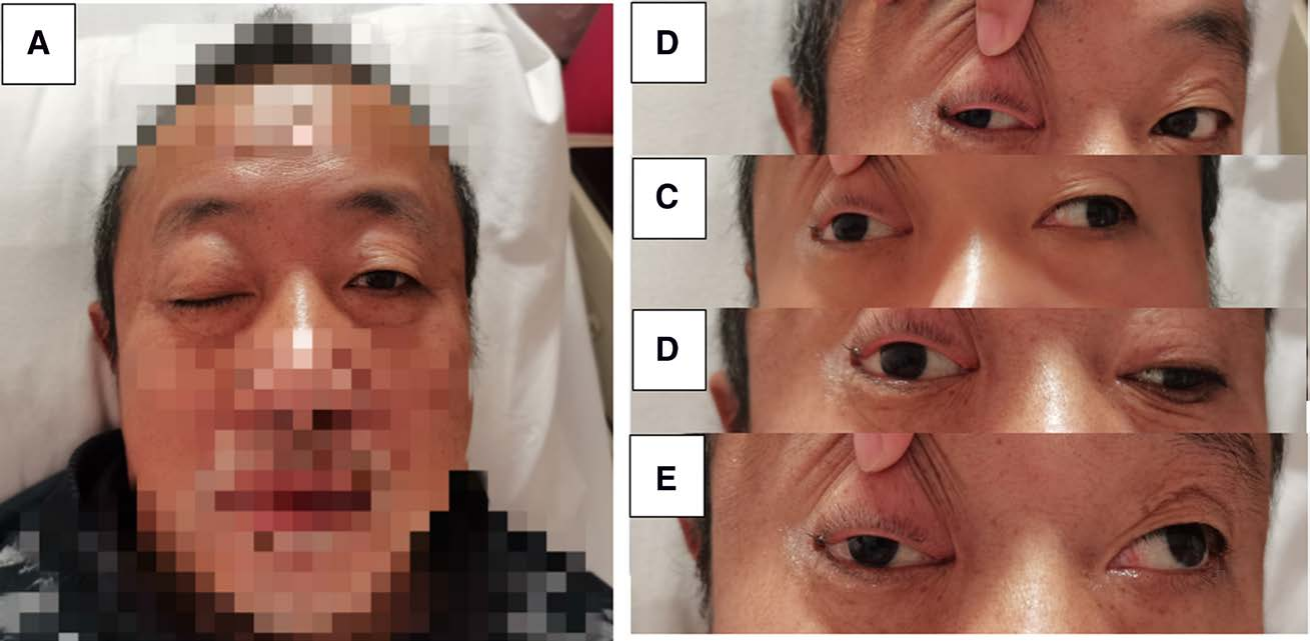
. Clinical manifestations of the patient (A)The patient had drooping right eyelid; (B) The patient may have abductive movement of the right eye; (C) The patient cannot do adduction in the right eye; (D) The patient cannot look down with the right eye; (E) The patient cannot look upward with the right eye.

The patient’s breathing, body temperature, and blood pressure were normal. On neurological examination, the patient was conscious and hoarse. The diameter of the left pupil was 0.25 cm, the diameter of the right pupil was 0.5 cm, the left eye was sensitive to light reflex, and the right eye had no direct and indirect light reflex. The movement of the left eye was normal, the right eye could only be abducted, could not be adducted, looked up, or looked down. Nystagmus was negative in both eyes (Fig. 1). The patient’s tongue was deviated to the right, and the uvula was deviated to the left. There was no edema in both lower extremities, muscle strength and muscle tension were normal, pharyngeal reflex was weakened. The patient’s tendon reflex symmetry was weakened. The bilateral Babinski sign was negative and bilateral depth sensation was normal.

**Figure 2.**
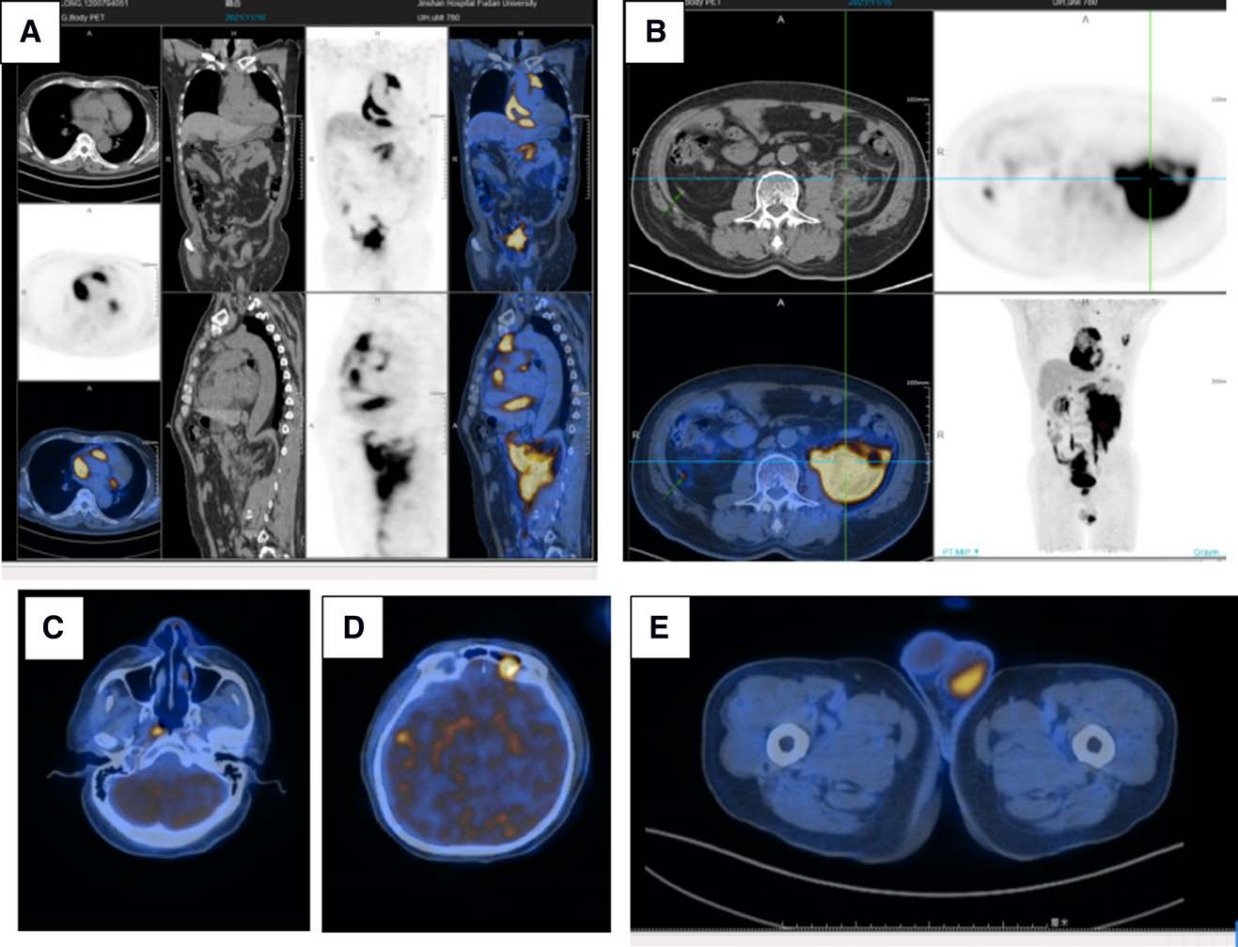
PET-CT scan images. (A–B). FDG uptake is increased in multiple areas of the body. (C). Abnormally elevated FDG uptake in the left orbital cavity; (D). Abnormally elevated FDG uptake in the left orbital cavity; (E). Abnormally elevated FDG uptake in the left testicle.

We have perfected a series of auxiliary inspections, including inflammatory markers, white blood cell and red blood cell counts, liver and kidney function indicators, tumor markers, electrolytes, cerebrospinal fluid tests, infectious disease-related antibody tests such as Treponema pallidum, HIV, HBV, etc. We added MRI enhancement and found abnormal signals in the center of semiovale and temporal lobe on both sides (Table 1). We also examined the indicators of paraneoplastic syndrome and autoimmune encephalitis in cerebrospinal fluid, and found no significant abnormalities. Orbital-enhanced CT showed no obvious abnormality. Although the patient’s common tumor indicators were not significantly abnormal, the patient’s LDH increased. Combined with the performance of the patient’s head MRI and the patient’s insensitivity to glucocorticoid therapy, we have a bad premonition. After communicating with the patient’s family, we decided to perform a Fluorine-18-fuorodeoxyglucose positron emission tomography-computed tomography (18F-FDG PET-CT) examination on the patient (Fig. 2). The results of PET-CT examination suggested an abnormal increase of glucose metabolism around bilateral lateral ventricles, choroid of temporal lobe, subependymal of fourth ventricle, left orbit, right pharynx, around atrium and aorta, anterior superior mediastinum, left axilla, bilateral perirenal fascia, peripancreatic body, right lower abdomen, pelvic cavity, prostate, and left testicle.

**Table 1:** Laboratory data.

Test item	Test value	Reference range
White blood cell count (10^9^/L)	8.3	3.5–9.5
Neutrophils (%)	86.1	40–75
Neutrophils (%)	7.4	20–50
Red blood cell count (10^9^/L)	3.98	4.3–5.8
Hemoglobin (g/L)	135	130–175
Lactate dehydrogenase (U/L)		
Syphilis	Negative	Negative
HIV-Ag/Ab	Negative	Negative
HBsAg	Negative	Negative
carcinoma embryonic antigen (ng/mL)	1.7	0–5
Neuron specific enolase (ng/mL)	12.53	0–16.3
Ferritin (ng/mL)	730.4	30–40
CSF examination		
CSF pressure (mm H2O)	150	80–180
CSF total cell count (10^6^/L)	55	0
CSF protein (g/L)	0.72	0.12–0.6
CSF glucose (mmol/L)	4.6	2.2–3.9
CSF IgG	27.4	0–34
CSF AQP4	Negative	Negative
CSF onconeural antibodies	Negative	Negative

AQP4 = aquaporin protein4, CSF = cerebrospinal fluid, HBsAg = hepatitis B surface antigen, HIV = human immunodeficiency virus, IGg = immunoglobulin G.

**Figure 3.**
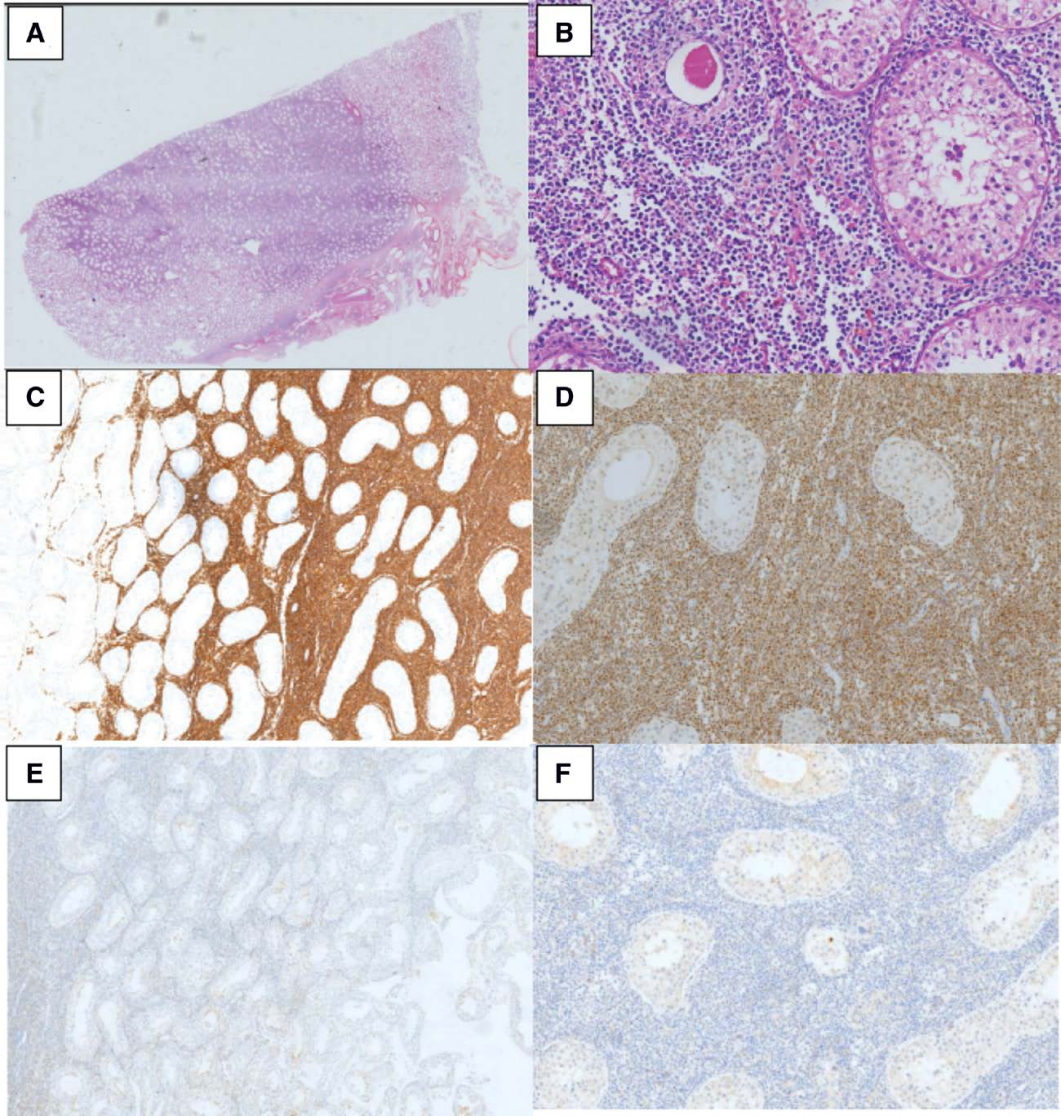
Pathological results of testicular biopsy specimen. (Fig A–B). hematoxylin and eosin (HE) staining. (Fig. A) The histopathology of testicular biopsy specimen showed dense proliferation of atypical lymphoid cells (HE, original magnification); (Fig. B) The nuclei are irregularly shaped and and concentration of nuclear chromatin (HE, 20*10); (Fig. C–F) Immunohistochemical (IHC) staining. Atypical lymphocytes were positive for CD20 (Fig. C, 4*10), MUM1 (Fig. D, 10*10), and BCL6 (Fig. E, 10*10) and negative for CD10 (Fig. F, 10*10). IHC = immunohistochemical, MUM-1 = multiple myeloma oncogene-1.

After communicating with the thoracic surgeon, urologist, ophthalmologist, and the patient’s family, we decided to perform left orchiectomy for safety reasons and sent for pathological examination. At this point, the patient becomes more advanced and drowsy, requiring food from an indwelling gastric tube. The patient developed respiratory alkalosis during the course of the disease. The patient’s pathologic findings suggested (left testicle) diffuse large B-cell lymphoma (non-GCB). Immunohistochemical (IHC) results of pathological tissue: CD20 (+); CD79*α* (+); CD3(-), CD7 (-), CD30 (-), CD117 (-), CK (-), *α*-inhibin (-), Vimentin (-/ +), PLAP (-), AFP (-), multiple myeloma oncogene-1 (MUM-1) (+), Bcl2 (+), CD43 (-), BCL6 (+), SOX11 (-), CD10 (-), CD21 (-), Ki67 (50%+), CD23 (-), CD5 (-) (Fig. 3). We pretreated with methylprednisolone 40 mg/day before chemotherapy according to the opinion of the hematologist. Informing the patient’s family that the patient’s current condition is progressing rapidly, the lymphoma has involved the brain, the patient’s general condition is poor, and central coma may occur, and the patient currently has symptoms of true bulbar palsy, weak cough reflex, and ventilatory dysfunction. The patient’s family decided to be discharged automatically. Unfortunately, 2 weeks after he was discharged, we called to follow up and were told the patient had passed away.

## 3. Discussion

We report a case of diffuse large B-cell lymphoma with oculomotor nerve palsy as the initial symptom. THS is a type of exclusion disease. When patients were first admitted to our hospital, there was a partial improvement in symptoms with glucocorticoids, which was very confusing. Therefore, corticosteroids should be used with caution in the diagnosis of central nervous system diseases. Especially for PCNSL, corticosteroid therapy may inhibit tumor growth and even lead to tumor regression, thus affecting the accuracy of diagnosis.^[11]^ In patients with painful oculomotor nerve palsy, in addition to common intracranial space occupyied such as aneurysm and venous thrombosis, the possibility of hematological malignancy should be considered. We believe that the patient’s oculomotor nerve palsy may be related to tumor involvement near the right cavernous sinus. Located on either side of the sella, the cavernous sinus is a sinus-like space consisting of 2 layers of dural membrane, which is actually an irregular network of small veins. The lateral wall of the cavernous sinus is composed of 2 layers, the outer layer is the continuation of the dura mater, and the inner layer is the nerve and sheath running on the lateral wall of the cavernous sinus. The oculomotor nerve (III), the trochlear nerve (IV), the first trigeminal nerve (V1) and the second trigeminal nerve (V2) are arranged from top to bottom between the 2 dural layers.^[12]^ Because in the anterior part of the cavernous sinus, the oculomotor nerve divides into the upper and lower branches, the lesions in this area may cause damage to the oculomotor nerve branches. When the first distribution of the trigeminal nerve is damaged, severe pain can occur in the forehead and around the orbit.

In previous studies, DLBCL with oculomotor nerve palsy was mainly due to primary central system lymphoma or orbital lymphoma directly invading the cavernous sinus region. Or the patient has a history of lymphoma and later metastases to the central nervous system. Through literature review, we did not find relevant reports similar to the case in this paper that oculomotor nerve palsy was the first symptom, there were no common lymphoma symptoms such as low fever and fatigue, and there was no history of blood system diseases, but multiple organs of the body were involved at the time of diagnosis. Since the patient had been involved in many organs of the whole body, and PET-CT showed that the patient was in immune sanctuary sites: CNS, testis, and eye all have lesions, which makes it very difficult to distinguish primary and secondary lesions. Previous studies have shown that PCNSL, as an aggressive lymphoma, has a median age of 56 years and is more likely to develop in men. PCNSL usually has a short course of disease, with a median survival time of less than 6 months.^[9]^ PTL is the most common testicular malignancy in men over 60 years of age, and is usually bilateral.^[7]^ Both PCNSL and PTL have a tendency to spread to other immunoprivileged sites. It is rare for lymphoma of the central nervous system, testicular, and orbital to be found at the same time. It is not clear whether lymphoma first appears in the nervous system and then spreads to multiple sites throughout the body, or if it appears at different sites at once. Although PCNSL rarely involves the whole body, combined with the pathogenesis of our patients, we are more inclined to think that this patient is PCNSL involving the whole body. Interestingly, Session 2 of the 2018 European Association of Hematopathology/ Society for Hematopathology Workshop, testicular involvement was found in all 3 cases of PCNSL mentioned in the workshop.^[13]^

IHC analysis of patients showed that b-lymphocyte membrane markers (CD20 and CD79a), Bcl-2, Bcl-6 and MUM-1 were all positive in tumor cells. The Ki67 IHC staining showed that the tumor cell proliferation index was 50%. PTL and PCNSL expressed pan-B cell antigens such as CD20, PAX5 and CD79a, and showed a non-GCB phenotype in most cases.^[14]^ The Ki67 index is a marker of cell proliferation. A higher Ki67 index predicts an aggressive tumor and therefore a poor prognosis. Moreover, studies have shown that the Ki67 index of non-GCB DLBCL tumor cells is significantly higher than that of GCB, indicating that the prognosis of non-GCB DLBCL is worse than that of GCB DLBCL.^[15]^ MUM-1 is a transcription factor in the interferon regulatory factor family, which is usually expressed in plasma cells and plays an important role in gene regulation. MUM-1 expression was closely associated with the ABC subgroup, suggesting poor overall survival and clinical response.^[16]^ Bcl-2 is an antiapoptotic protein that primarily protects cells. Its expression may be involved in carcinogenesis and tumor resistance to antitumor drugs, often indicating a poorer prognosis. Bcl-6, a transcription repressor involved in germinal center formation, also predicted poor overall survival.^[17]^ Unfortunately, we cannot determine the results of MYC protein expression. If MYC is positive on immunohistochemistry, it is a triple-hit lymphoma with a very poor prognosis.

## 4. Conclusion

In conclusion, simultaneous lymphomas in multiple parts of the body, especially those involving more than 1 immunoprivileged site, are very rare. Atypical clinical symptoms tend to confuse the doctor’s diagnosis. THS is an exclusive diagnosis. In order to avoid masking the true condition, adequate examination should be performed before glucocorticoid therapy. Early improvement of PET-CT examination may lead to early detection of lesions and better prognosis.

## Author contributions

**Writing – original draft:** Zhang Yimei.

**Writing – review & editing:** Zhang Jianfeng.
